# The Emerging Roles of circFOXO3 in Cancer

**DOI:** 10.3389/fcell.2021.659417

**Published:** 2021-06-04

**Authors:** Dean Rao, Chengpeng Yu, Jiaqi Sheng, Enjun Lv, Wenjie Huang

**Affiliations:** ^1^Hepatic Surgery Center, Tongji Hospital, Tongji Medical College, Huazhong University of Science and Technology, Wuhan, China; ^2^Hubei Key Laboratory of Hepato-Pancreato-Biliary Diseases, Tongji Hospital, Tongji Medical College, Huazhong University of Science and Technology, Wuhan, China

**Keywords:** circular RNA, circFOXO3, cancer, microRNA sponge, biomarker

## Abstract

Circular RNAs (circRNAs) are a class of endogenous non-coding RNAs which are mainly formed by reverse splicing of precursor mRNAs. They are relatively stable and resistant to RNase R because of their covalently closed structure without 5’ caps or 3’ poly-adenylated tails. CircRNAs are widely expressed in eukaryotic cells and show tissue, timing, and disease specificity. Recent studies have found that circRNAs play an important role in many diseases. In particular, they affect the proliferation, invasion and prognosis of cancer by regulating gene expression. CircRNA Forkhead box O3 (circFOXO3) is a circRNA confirmed to be abnormally expressed in a variety of cancers, including prostate cancer, hepatocellular carcinoma, glioblastoma, bladder cancer, and breast cancer, etc. At present, the feature of circFOXO3 as a molecular sponge is widely studied to promote or inhibit the development of cancers. However, the diverse functions of circFOXO3 have not been fully understood. Hence, it is important to review the roles of circFOXO3 in cancers. This review has summarized and discussed the roles and molecular mechanism of circFOXO3 and its target genes in these cancers, which can help to enrich our understanding to the functions of circRNAs and carry out subsequent researches on circFOXO3.

## Introduction

Circular RNAs (circRNAs) are a special class of non-coding RNA. Different from traditional linear RNA, circRNAs have covalently closed circular structure and lack 5′-3′ polarity or 3′ poly-adenylated tails (Sanger et al., 1976; Jeck et al., 2013). In the beginning, circRNAs were rarely reported by scientists as being low in abundance and might represent splicing errors because of the error-prone mechanism of exon juxtaposition (Bailleul, 1996; Jeck et al., 2013). With the development of high-throughput sequencing technology, circRNAs were discovered in large quantities. They are abundant in various eukaryotic organisms and have certain tissue, timing and disease specificity, which implies that the expression of circRNAs is related to the cellular microenvironment (Salzman et al., 2013; Rybak-Wolf et al., 2015; Han et al., 2018). Moreover, circRNAs have been found in blood, saliva and other body fluids (Bahn et al., 2015; Preusser et al., 2018; Li Y. et al., 2019), even many exosomes have higher ratio of circRNAs than cells (Li Y. et al., 2015). Furthermore, circRNAs are resistant to the degradation of exonuclease RNase R due to their special closed circular structure, which makes them greater stability and longer half-life (Jeck et al., 2013; Chen, 2016; Panda et al., 2017; Xiao and Wilusz, 2019). These characteristics and the development of bioinformatics make circRNAs popular biomarkers for disease diagnosis and the research on drug therapeutic targets.

Circular RNAs are mainly classified into three types: exonic circRNAs (EcircRNAs), exon-intron circRNAs (ElciRNAs), and circular intronic RNAs (ciRNAs). EcircRNAs exist in cytoplasm and can regulate gene expression by limiting the roles of miRNAs (Salzman et al., 2012; Hansen et al., 2013). ElciRNAs and ciRNAs mainly exist in nucleus to act as transcription regulators (Zhang et al., 2013; Li Z. et al., 2015). In addition, it has also been reported that viral RNA genome, transfer RNA, ribosomal RNA, and small nuclear RNA can be cyclized into circRNAs (Lasda and Parker, 2014; Schmidt et al., 2019). CircRNAs have numerous biological functions, including: (1) MiRNAs sponge. CircRNAs have complementary miRNAs binding sites and can competitively bind miRNAs to inhibit their functions. This mechanism is the focus of current research and mainly exercised by ecircRNAs (Hansen et al., 2013; Li R. et al., 2020). (2) Combining with RNA-binding proteins (RBPs). CircRNAs can competitively bind with RBPs to regulate the function of RBPs and have an influence on mRNA stability and splicing patterns (Zang et al., 2020). (3) Regulating Transcription. Although ecircRNAs, as a major part of circRNAs, play an important role in the cytoplasm, elciRNAs and ciRNAs are mainly located in the nucleus and can regulate gene transcription by combining with RNA polymerase or other transcription-related components (Hansen et al., 2013; Chen, 2016). (4) Translation. Some studies demonstrate that circRNAs are associated with translation of ribosomes and N6-Methyladenosine can drive extensive translation of circRNAs (Legnini et al., 2017; Pamudurti et al., 2017; Yang et al., 2017). (5) Interacting with proteins. For example, CircRNA Forkhead box O3 (circFOXO3) functions as a dynamic scaffolding molecule that regulates the interaction between cyclin-dependent kinase 2 (CDK2) and cyclin-dependent kinase inhibitor 1 (P21; Du et al., 2016, 2017b). CircACC1 can directly bind to the β and γ subunits of AMPK, promoting its stability and activity (Li Q. et al., 2019). (6) Functions in exosomes. CircRNAs can enter body fluids under the protection of exosomes to transmit biological information and substances to target cells, and regulate cell growth, epithelial mesenchymal transformation, angiogenesis, and other aspects (Wang Y. et al., 2019; Shi et al., 2020). In conclusion, circRNAs have various functions and participate in the regulation of physiological activities through different pathways. In recent years, circRNAs have often been used in basic research and bioinformatics analysis, reflecting the potential of circRNAs as biomarkers (Li Y. et al., 2019; Wang J. et al., 2020).

Circular RNAs are closely related to the occurrence and development of various human diseases (Aufiero et al., 2019; D’Ambra et al., 2019; Liu et al., 2019; Haque et al., 2020; Li R. et al., 2020). In particular, circRNAs are present in cancer diagnosis, development, drug resistance and circFOXO3 is one of the important ones (Zhang and Xin, 2018; Greene et al., 2019; Chen et al., 2020). The FoxO subfamily of forkhead transcription factors (Fox) widely exists in eukaryotic cells and is involved in the regulation of cell cycle, energy metabolism and tumorigenesis through specific activation of transcription process (Link, 2019). The mammalian system consists of four members, FOXO1, FOXO3, FOXO4, and FOXO6, which are regulated by the PI3K-PKB signaling pathway (Liu et al., 2018). Among them, FOXO3 is widely expressed and highly correlated with a series of malignant tumors such as breast cancer, prostate cancer (PCa) and acute myeloid leukemia (AML; Du et al., 2017a; Zhou et al., 2019; Kong et al., 2020). CircFOXO3 is formed from the exon 2 of FOXO3 and it can not only increase the protein level of FOXO3, but also participate in the post-transcriptional regulation of transcription products through the competitive endogenous RNAs (ceRNAs) network, thus having a dual effect on the development of cancers (Tay et al., 2014; Du et al., 2017a; Zhou et al., 2019). To sum up, the functions of circFOXO3 are complex and important for cancer research. In the following chapters, we will summarize the characteristics of circFOXO3 and its role in cancer through existing studies, so as to provide some basic knowledge for subsequent studies and some inspiration for its research direction in cancers.

## The Biological Functions of circFoxo3

**FIGURE 1:**
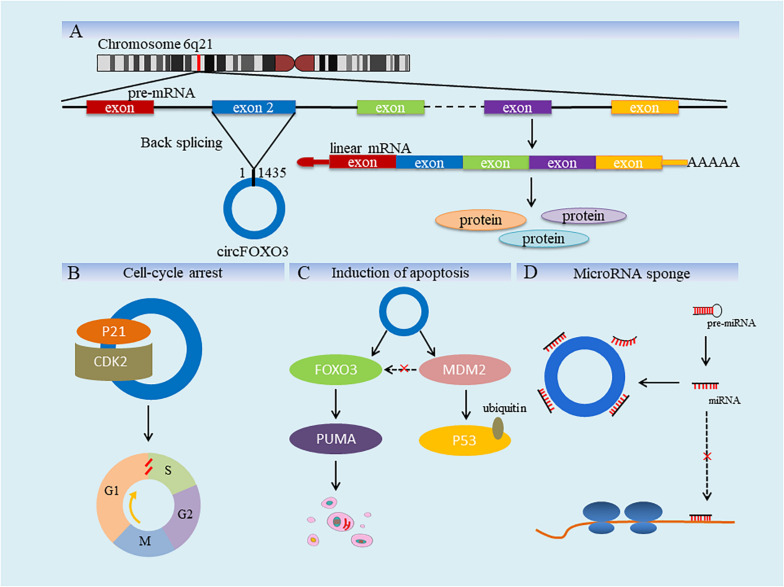
Diagram for biogenesis and functions of circFOXO3. **(A)** As shown, FOXO3 is located in chromosome 6q21 in human, and the red line is used to represent the approximate position of it. FOXO3 contains nine exons and only part of them is shown in this figure. circFOXO3 is formed by reverse splicing of exon 2, containing 1435 nucleotides (chr6:108,984,657–108,986,092). **(B)** CircFOXO3 forms ternary complex by combining CDK2 and P2 and the cell cycle is blocked in S phase. **(C)** CircFOXO3 promotes the regulation of FOXO3 on apoptosis by reducing FOXO3 ubiquitination. **(D)** CircFOXO3 adsorbs miRNAs and weakens the negative effect of the latter on gene expression.

CircRNA Forkhead box O3 is a closed circular RNA that contains 1435 nucleotides, formed from the exon 2 of FOXO3 gene (Figure 1A) and the biological functions of it overlap with that of FOXO3 partly. Just like other circRNAs, circFOXO3 has extensive and complex biological functions, which are currently known to be related to cell differentiation, apoptosis and cell cycle progression. For example, Li et al. found that the expression of myogenin (MyoG) and myosin heavy chain (MyHC) was significantly increased by interfering with the expression of circFOXO3. MyoG and MyHC are important marker genes for muscle differentiation, and the effect of circFOXO3 on them can inhibit the differentiation of myoblast cells (Li X. et al., 2019). Furthermore, the overexpression of circFOXO3 is also associated with glutamate-induced oxidative damage in HT22 cell line (Hippocampal neurons from mice). Silencing circFOXO3 can protect HT22 cells by reducing the loss of glutamate-induced mitochondrial membrane potential (Lin et al., 2020). In terms of the effect on cell cycle, circFOXO3 forms ternary complex by combining with CDK2 and P21 (Figure 1B). CDK2 can promote the entry of cell cycle by interacting with cyclin A and cyclin E, while p21 has an opposite effect on cell cycle (Bivik Stadler et al., 2019). CircFOXO3-p21-CDK2 ternary complex blocks the formation of cyclin E/CDK2 complex and eliminates the inhibition of cyclin A/CDK2 complex by p21. As a result, cell cycle is blocked in G1 phase and the process is delayed (Du et al., 2016). By the way, it has also been observed that circFOXO3 in peripheral blood is specifically expressed in the elderly compared to young people, which is related to cellular senescence and has certain predictive significance for human senescence phenotype (Haque et al., 2020).

Sun et al. (2018) fount that ipatasertib, a novel ATP-competitive pan-AKT inhibitor, was used in the treatment of colon cancer to inhibit AKT activity, and then FOXO3 was activated, which up-regulated p53 up-regulated modulator of apoptosis (PUMA), leading to PUMA/Bax-dependent endogenous apoptosis, thereby exerting the anticancer effect of ipatasertib. Similarly, Du et al. (2017a) found that the overexpression of circFOXO3 inhibited the interaction between FOXO3 and mouse double minute 2 (MDM2), prevented MDM2 from inducing ubiquitination and degradation of FOXO3, thus increasing the activity of FOXO3 and promoting the expression of PUMA to induce apoptosis of cancer cells (Lin et al., 2020; Figure 1C). In addition, the main function of circRNAs in cancers is to influence the post-transcriptional regulation of other genes by acting as miRNA sponge (Figure 1D). Using database such as RegRNA 2.0 or Circinteractome, it is very convenient to predict miRNA binding sites through circFOXO3 sequence and some of them have been experimentally confirmed (Dudekula et al., 2016; Liu et al., 2016). For example, circFOXO3 promotes solute carrier family 25 member 15 (SLC25A15) transcription by acting as a miR-29a-3p sponge, affecting the apoptosis and cell cycle of PCa and showing carcinogenic activity (Kong et al., 2020). In glioblastoma (GBM), circFOXO3 also plays a pro-tumor role to adsorb both miR-138-5p and miR-432-5p. It is worth mentioning that the inhibition of circFOXO3 downregulation on GBM can be reversed by miR-138-5p and miR-432-5p inhibitors (Zhang et al., 2019).

## The Roles of circFoxo3 in Cancers (Figure 2 and Table 1)

### Prostate Cancer

Prostate cancer is an epithelial malignant tumor occurring in the prostate gland. It was reported that circFOXO3 was up-regulated in PCa tissues and serum samples and played a tumor-promoting role (Kong et al., 2020; Li et al., 2021). Moreover, Li et al. (2021) proved that circFOXO3 was stable in PCa through RNase R digestion and Actinomycin D exposure. Silencing circFOXO3 could significantly inhibit the development of PCa in many aspects. For example, knockdown of circFOXO3 affected the normal cell cycle of PCa cells and reduced cell viability, thereby inhibiting tumor proliferation and invasion. At the same time, the apoptosis rate of tumor cells increased, reflecting the effect of circFOXO3 in inhibiting the apoptosis of PCa cells (Kong et al., 2020; Li et al., 2021). Interestingly, they found different mechanisms by which circFOXO3 promoted the development of PCa. First, circFOXO3 could target miR-29a-3p in PCa cells and enhance the expression level of SLC25A15 (Kong et al., 2020). Li et al. (2021) demonstrated that circFOXO3 acted as a miR-1299 sponge to up-regulate the expression of cofilin 2, thus showing carcinogenic activity. In addition, Shen et al. (2020) stated contradictory research conclusion. They suggested that circFOXO3 inhibited the progression of PCa by increasing the level of linear FOXO3 and the reduction of circFOXO3 promoted chemotherapy resistance of docetaxel. This discrepancy may stem from differences in the handling of clinical samples and the cell lines used in the laboratory. More experiments are needed to confirm it.

**FIGURE 2:**
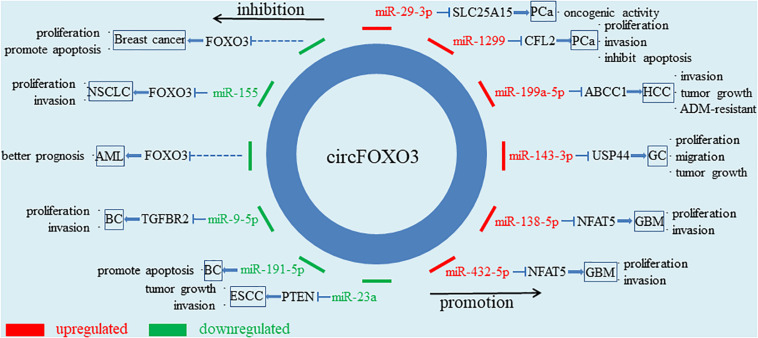
Diagram for circFOXO3 acts as a miRNA sponge. CircFOXO3 functions as a miRNA sponge to adsorb multiple miRNAs, thus regulating the expression of downstream genes and cancer progression.

**TABLE 1:** The expression and roles of circFOXO3 in cancers.

**Cancer type**	**Expression in cancers**	**Related miRNAs and genes**	**Functional roles**	**References**
Prostate cancer (PCa)	Up-regulated	miR-29-3p/SLC25A15	Exhibit oncogenic activity	Kong et al., 2020
		miR-1299/CFL2	Promote cell proliferation, invasion. Inhibit apoptosis	Li et al., 2021
	Down-regulated	–	Enhance cancer viability, progression and chemoresistance to docetaxel	Shen et al., 2020
Hepatocellular Carcinoma (HCC)	Up-regulated	miR-199a-5p/ABCC1	Promote cell invasion, tumor growth and ADM-resistant	Huang et al., 2020
Gastric carcinoma (GC)	Up-regulated	miR-143-3p/USP44	Promote cell proliferation, migration and tumor growth	Xiang et al., 2020
Glioblastoma (GBM)	Up-regulated	miR-138-5p, miR-432-5p/NFAT5	Promote proliferation and invasion	Zhang et al., 2019
Esophageal squamous cell cancer (ESCC)	Down-regulated	miR-23a/PTEN	Inhibit cell growth and invasion	Xing et al., 2020
Bladder cancer (BC)	Down-regulated	miR-191-5p	Promote cell apoptosis	Wang C. et al., 2019
		miR-9-5p/TGFBR2	Inhibit cell proliferation and invasion	Li Y. et al., 2020
Acute myeloid leukemia (AML)	Down-regulated	FOXO3	Lead to better prognosis	Zhou et al., 2019
Non-small cell lung cancer (NSCLC)	Down-regulated	miR-155/FOXO3	Inhibit proliferation and invasion	Zhang et al., 2018
Breast cancer	Down-regulated	FOXO3	Inhibit proliferation. Promote apoptosis	Du et al., 2017a

### Hepatocellular Carcinoma

Hepatocellular carcinoma (HCC) is one of the most common malignant tumor worldwide, with a high mortality rate and a serious threat to human health (Craig et al., 2020). Huang et al. (2020) reported the expression, role and mechanism of circFOXO3 in HCC detailedly. Increased expression of circFOXO3 was detected in HCC cells and metastatic tissues. The overexpression of circFOXO3 promoted cancer growth through miR-199a-5p/ATP binding cassette subfamily C member 1 (ABCC1) pathway, which was manifested in the increase of cancer volume and average cancer mass. Epithelial-mesenchymal transition (EMT) is an essential process for the invasion and metastasis of epithelial-cell derived malignancies, and circFOXO3 can promote EMT by interacting with miR-199a-5p (Giannelli et al., 2016; Huang et al., 2020). In addition, Huang et al. emphasized that the expression of circFOXO3 was increased in adriamycin-resistant HCC tissues. Chemoresistance is based on intratumoral heterogeneity and is affected by many factors such as cancer microenvironment and intracellular gene expression (He et al., 2016; Yeldag and Rice, 2018). CircFOXO3 promoted adriamycin resistance in HCC by relieving the restriction effect of miR-199a-5p on ABCC1 (Huang et al., 2020). In conclusion, highly expressed circFOXO3 in HCC cells, especially in adriamycin-resistant HCC tissues, could promote the proliferation, invasion and drug resistance of HCC through miR-199a-5p/ABCC1 axis, and indicated a higher degree of malignancy and a poorer prognosis.

### Gastric Carcinoma

The role of circFOXO3 in gastric cancer (GC) is also realized through the ceRNAs mechanism. Xiang et al. (2020) found that the overexpressed circFOXO3 interacted with miR-143-3p to limit its function, and then the expression of ubiquitin-specific protease 44 (USP44) was up-regulated. USP44, which belongs to USP family, can induce chromosome instability, resulting in DNA aneuploidy in GC (Zhang et al., 2011; Nishimura et al., 2017). Subsequently, experiments *in vivo* and *in vitro* showed that circFOXO3 promoted the progression of GC, including proliferation and migration of GC cells. In conclusion, circFOXO3 promoted the malignant progression of GC through the miR-143-3p/USP44 axis (Xiang et al., 2020). Considering the important role of USP44 in the development of GC, it is of great clinical significance to explore the targeting effect of circFOXO3 in GC treatment.

### Glioblastoma

Glioblastoma, originating from the neuroepithelium, is the most common intracranial malignancy (Tan et al., 2020). Similar to the above, circFOXO3 played an important role in the occurrence and progression of GBM. CircFOXO3 was not only up-regulated in GBM cells, but also correlated with the histological grade of GBM. The expression of circFOXO3 in low grade GBM was obviously inferior to that in high grade GBM (Zhang et al., 2019). What’s more, GBM can be divided into isocitrate dehydrogenase (IDH) wild type and IDH mutant type at the gene molecular level, and the efficacy of chemotherapy in GBM patients is closely related to the methylation state of O6-methylguanine-DNA methyltransferase (MGMT; Miller et al., 2017; Schaff et al., 2020). Zhang et al. (2019) proved that the expression of circFOXO3 was also significantly associated with wild-type IDH expression and MGMT methylation. They found that miR-138-5p and miR-432-5p jointly targeted nuclear factor of activated T cells 5 and restricted its expression, while circFOXO3 could remove this restriction and promote the proliferation and invasion of GBM cells (Zhang et al., 2019). At the same time, Chen et al. (2020) showed that plasma circFOXO3 was highly expressed in patients with GBM and had predictive significance for the occurrence of GBM.

### Esophageal Squamous Cell Cancer

According to the pathological classification, esophageal carcinoma is mainly divided into squamous cell carcinoma, adenocarcinoma and other less common types. At present, it has been reported that circFOXO3 negatively regulates the progression of esophageal squamous cell carcinoma (ESCC). Compared with paracancerous tissues and normal esophageal endothelial cells, circFOXO3 was down-regulated in ESCC tissues and various cell lines (KYSE510, TE-1, TE-13, and ECA109; Xing et al., 2020). Overexpression of circFOXO3 inhibited the development of ESCC, including reduced proliferation and invasion of tumor cells. At the same time, increased cells in G0/G1 phase and decreased cells in S phase were observed. CircFOXO3 promoted apoptosis and cell cycle arrest through miR-23a/PTEN axis, thereby inhibiting the progression of ESCC *in vivo* and *in vitro* (Xing et al., 2020). MiR-23a could promote the proliferation of ESCC cells and improve cell viability. Another study also showed that high plasma level of miR-23a was associated with the progression of ESCC and could be used as an independent risk factor in ESCC patients (Komatsu et al., 2016). As for phosphatase and tensin homolog (PTEN), which is known as an important tumor suppressor and metabolic regulator, it is the ultimate mechanism for the effect of circFOXO3 in cancer (Milella et al., 2015; Chen et al., 2018). Understanding the metabolic regulation mechanism of PTEN is of great significance for the treatment of ESCC.

### Bladder Cancer

Li Y. et al. (2020) found a series of down-regulated circRNAs in bladder cancer (BC) tissues and circFOXO3 was one of them. BC was negatively regulated by upregulating the expression of circFOXO3. In order to clarify the underlying mechanism, they used bioinformatics analysis and found the miR-9-5p/transforming growth factor beta receptor 2 (TGFBR2) pathway. Experiments proved that over-expressed circFOXO3 could up-regulate TGFBR2, a key protein in TGF-β signaling pathway, through the interaction with miR-9-5p and finally regulate the proliferation and invasion of BC cells (Li Y. et al., 2020). Moreover, Wang C. et al. (2019) showed that circFOXO3 was lowly expressed in BC tissues *in vivo* and *in vitro*, which was consistent with the findings of another set of experiments. However, when doxorubicin, cisplatin, or H_2_O_2_ were used to promote BC cell apoptosis, the expression of circFOXO3 was up-regulated. In BC cell lines and mice BC tissues, circFOXO3 did induce apoptosis of cancer cells. It was further found that the pro-apoptotic ability of circFOXO3 was inhibited by miR-191-5p (Wang C. et al., 2019). The pathway by which miR-191-5p regulates circFOXO3 has not been thoroughly explained. However, some miRNAs are reported to regulate circRNAs in an AGO2-dependent way (Hansen et al., 2011; Pan et al., 2019). There may be some similar mechanisms for this process.

### Acute Myeloid Leukemia

Acute myeloid leukemia is a malignant clonal disease of myeloid hematopoietic stem/progenitor cells. Zhou et al. reported that the expression level of circFOXO3 in AML patients was lower than that of normal people and had certain diagnostic value. Moreover, bioinformatics analysis of the expression levels of circFOXO3 and FOXO3 in AML cell lines showed a positive correlation (Zhou et al., 2019). Through the analysis of FOXO3-related experiments, the benign effect of circFOXO3 on the prognosis might be related to the promotion of AML cell apoptosis by FOXO3 (Sakoe et al., 2010; Li J.X. et al., 2019). Of course, more experiments are needed to confirm the exact mechanism.

### Non-Small Cell Lung Cancer

Lung cancer can be divided into small cell lung cancer (SCLC) and non-small cell lung cancer (NSCLC) according to the morphology of tumor cells. NSCLC accounts for about 80% of all lung cancers, with a high degree of malignancy and a low 5-year survival rate. CircFOXO3 was down-regulated in both NSCLC cell lines and tissues and exhibited tumor suppressive effects (Zhang et al., 2018). Similar to its role in other cancers, circFOXO3 regulated downstream gene expression by adsorbing miRNAs. However, the downstream gene of miR-155 was exactly the linear isomer of circFOXO3 (Zhang et al., 2018). The regulation of FOXO3 by miR-155 was related to oral cancer, HCC, nasopharyngeal cancer and many other cancers (Liao et al., 2018; Wu et al., 2019; Li X. et al., 2020). In NSCLC, the proliferation and invasion ability of tumor cells were weakened after FOXO3 was activated without the restriction of miR-155 (Zhang et al., 2018). MiR-155/FOXO3 axis has a wide range of effects and plays an important role in the development of NSCLC, suggesting a promising therapeutic target for NSCLC.

### Breast Cancer

Du et al. found that circFOXO3 was low expressed in breast cancer tissues and cell lines, but up-regulated in apoptotic cancer cells. The high expression of circFOXO3 could not only inhibit tumor growth, but also significantly increase the apoptosis of tumor cells transfected with circFOXO3 (Du et al., 2017a). MDM2 is a negative regulator of P53, which can induce P53 ubiquitination to promote cancer (Wade et al., 2013; Du et al., 2017a; Wang W. et al., 2020). Furthermore, Du et al. detected that MDM2 could promote the ubiquitination of FOXO3 and P53 in breast cancer. Although circFOXO3 had little effect on the expression of MDM2, it could enhance the interaction between MDM2 and P53, thus weakening the ubiquitination of MDM2 on FOXO3. Ultimately, FOXO3 activated its downstream target gene, PUMA, and promoted apoptosis (Du et al., 2017a).

## The Potential of circFoxo3 as a Prognostic Factor in Cancers

CircRNA Forkhead box O3 plays an important role in the occurrence, progression, and prognosis of many cancers. Understanding the expression pattern of circRNAs in cancers is of great significance for predicting the prognosis of patients. However, the expression of circFOXO3 varies greatly in different cancers. For example, circFOXO3 is up-regulated in HCC, GC, and GBM, but down-regulated in ESCC, BC, AML, NSCLC, and breast cancer. The exact reasons for the discrepancy are unclear. This difference may be related to the timing and tissue specificity of circRNAs (Salzman et al., 2013). As Li X. et al. (2019) found in mice, circFOXO3 was most expressed in hearts and least expressed in the kidneys among all organs. Moreover, it is known that circFOXO3 is formed by reverse splicing of the exon 2 of FOXO3, and the regulatory mechanism in this process hasn’t been mentioned yet. There may be some regulatory process that affects the expression of circFOXO3 in cancers. In addition, circFOXO3 is inhibited by miRNAs. MiRNAs can promote the splicing or cleavage of circFOXO3, which also has certain influence on the expression of circFOXO3 in cells (Hansen et al., 2011; Pan et al., 2019). Furthermore, circFOXO3 has its own characteristics in various cancers and is related to many clinical features of patients. The expression of circFOXO3 in PCa is correlated with Gleason score and chemotherapy resistance (Kong et al., 2020; Shen et al., 2020). In GBM, the expression level of circFOXO3 in high-grade tumor tissues is significantly higher than that in low-grade tumor tissues, which suggests a poor prognosis (Zhang et al., 2019). What’s more, circFOXO3 is sensitive to the state of tumor cells, and may exhibit an opposite expression state when stimulated by apoptosis (Du et al., 2017a; Wang C. et al., 2019). In addition, although peripheral blood tests show specific expression of circFOXO3 in the elderly, the expression of circFOXO3 in tumors does not appear to be affected by age (Haque et al., 2020; Kong et al., 2020). These evidences suggest that circFOXO3 has sufficient sensitivity and specificity for the clinical status of patients and can be a promising biomarker.

## Conclusion

For a long time, scientists have never stopped searching for a cure for cancers and the discovery of circRNAs, in particular, has provided scientists with a new way to tackle cancers. Among them, circFOXO3 is extensively studied for its diverse functions in cancers. Overall, the expression of circFOXO3 varies among different types of cancer, and even the expression level detected in the same type of cancer remains controversial. However, it is clear that circFOXO3 is involved in the development of cancers by regulating the expression of multiple downstream genes. In addition to affecting the apoptosis, proliferation, migration and invasion of cancer cells, circFOXO3 is also associated with clinical characteristics, chemotherapy resistance, etc. Since the roles of circFOXO3 in different cancers are not consistent, which poses a challenge for circFOXO3 as a potent biomarker, a timely summary of the expression and roles of circFOXO3 in these cancers is necessary.

At present, our understanding of circFOXO3 is insufficient due to the lack of repeated experiments and additional samples. Insufficient experimental data and researchers’ overemphasis on some downstream genes may obscure the truth. Therefore, more experiments are needed to determine the expression characteristics of circFOXO3 in various cancers and at different stages of the same cancer. At the same time, we need to explore a deeper and broader functional mechanism of circFOXO3, which is conducive to discover the dominant mechanism by which circFOXO3 acts on cancer. Furthermore, referring to the roles of circFOXO3 in non-neoplastic diseases, such as its effect on heart disease, may be useful to our exploration of cancer. In conclusion, according to the types of cancer, circFOXO3 is a potential and specific biomarker for predicting the occurrence of cancers and guiding clinical practice. With the development of the technology and the efforts of the researchers, it is expected to discover its potential value and contribute to the treatment of cancer.

## Author Contributions

DR drafted and revised the manuscript. WH designed the review. CY, JS, and EL participated in the design of the review and helped to draft and revise the manuscript. All authors read and approved the final manuscript.

## Conflict of Interest

The authors declare that the research was conducted in the absence of any commercial or financial relationships that could be construed as a potential conflict of interest.
